# Results of day-case ureterorenoscopy (DC-URS) for stone disease: prospective outcomes over 4.5 years

**DOI:** 10.1007/s00345-017-2061-1

**Published:** 2017-06-15

**Authors:** Anngona Ghosh, Rachel Oliver, Carolyn Way, Lucy White, Bhaskar K. Somani

**Affiliations:** 1 Department of Urology, University Hospital Southampton NHS Trust, Southampton, SO16 6YD UK; 2 Department of Anaesthetics, University Hospital Southampton NHS Trust, Southampton, SO16 6YD UK

**Keywords:** Day case, Ureteroscopy, Ureterorenoscopy, Stones, Urolithiasis, Laser, Kidney

## Abstract

**Purpose:**

To investigate the prospective outcomes of day-case ureterorenoscopy (DC-URS) for stone disease. With the rising prevalence of stone disease in the face of finite resources, there is increasing pressure to undertake procedures as a day case avoiding in-patient stay. There are a limited number of studies reporting on the feasibility of ureteroscopy as a day-case procedure. This study aimed to investigate the prospective outcomes and predictors precluding to DC-URS for stone disease in patients treated in our university teaching hospital.

**Materials and methods:**

Between March 2012 and July 2016, consecutive cases of adult stone ureteroscopy performed or supervised by a single surgeon were recorded in a prospective database. Patients underwent pre-operative counselling in a specialist stone clinic and were admitted to a dedicated ‘Surgical day unit’ on the day of surgery. A standardised anaesthetic protocol was adhered to in all cases. Data on patient demographics, stone parameters, pre-operative assessment, operative details, length of stay, stone-free rate and complication rates were collected and analysed.

**Results:**

A total of 544 consecutive adult ureteroscopy for stone disease were conducted over the study period with a day-case rate of 77.7%. Thirty-nine percent of failed day-case ureteroscopy were due to late completion of ureteroscopy and due to associated social circumstances of patients. The mean stone size, operating time duration and post-operative stent insertion rates for DC-URS patients were 14 mm, 46 min and 96.5%, respectively. Post-operatively, the mean stone-free rate (SFR), unplanned re-admissions and complications for DC-URS patients were 95, 4 and 4%, respectively. A higher failure of DC-URS was related to patient’s age (*p* = 0.003), positive pre-operative urine culture (*p* < 0.001), elevated pre-operative serum creatinine (*p* < 0.001) and higher mean operating time (*p* < 0.02).

**Conclusion:**

Based on our results, a day-case ureteroscopy rate of nearly 78% can be achieved. With its acceptable complication rate, and low re-admission rates, DC-URS is a safe and feasible option in a majority of patients with stone disease.

## Introduction

We are witnessing an ever increasing rise in the incidence of stone disease and with a lifetime prevalence of almost 14%, this has direct implications for patient morbidity and demand on healthcare resources [1, 2]. In the last few decades, there has been a dramatic increase in the use of ureteroscopy (URS) with widening of its indications, now becoming a routine urological procedure [2–7]. Being an endoscopic procedure with minimal risk of significant bleeding and a relatively short operating time, URS lends itself to being a suitable treatment modality as a day-case procedure, with studies demonstrating its feasibility as an outpatient procedure [8–10]. In the face of reduced resources, there is rising pressure for procedures to be undertaken on a day-case basis avoiding in-patient stay [1, 2]. Hence, there is no doubt that same day discharge of patients undergoing stone treatment is an attractive option allowing for financial benefits, potential for improved efficiency by healthcare providers and greater patient satisfaction [3, 8, 9]. The objective of this study is to investigate the prospective outcomes and predictors of failure of day-case URS (DC-URS) at our university teaching hospital.

## Materials and methods

All adults who underwent ureteroscopy (URS) procedure for stone disease over a 52-month period between March 2012 and July 2016 were included in this study. Our audit was registered with our hospital ‘Clinical Effectiveness (CE) and Audit office’ with patient consent for participation taken prior to the procedure. Outcomes were collated prospectively for consecutive adults (>18 years of age) who underwent URS for stone disease, performed or supervised by a single surgeon (BS) at our institution and recorded in our prospective database which was then analysed retrospectively by a third party (AG, RO) not involved in the original procedure. Data were collected for patient demographics, stone parameters, pre-operative assessment, operative details, length of stay (LoS), stone-free rate (SFR) and complication rates associated with the procedure. LoS was defined as the time from completion of URS to discharge of patients with day case defined as those patients discharged on the same day as having undergone the procedure. Data were recorded on excel spread sheet and analysed using SPSS version 22.

### Pre-operative assessment

Stone diagnosis was confirmed using non-contrast abdominal computed tomography kidney–ureter–bladder (CT KUB) and all patients underwent counselling for their URS procedure in a specialist kidney stone clinic. All patients had an anaesthetist led pre-assessment and were counselled for a day-case procedure unless there were anaesthetic or surgical concerns necessitating an in-patient stay. Pain relief was discussed in order to set expectations about the level of post-operative pain. Patients with a positive urine culture were given appropriate sensitivity-based antibiotics with a repeat urine culture arranged.

### Use of an anaesthetic protocol

On the day of surgery, patients were admitted to our dedicated ‘Surgical Day Unit’ where they met the anaesthetist and treating surgeon for assessment, counselling and consent for the procedure. A laryngeal mask airway with spontaneous breathing was used as the default for airway management. All patients were discharged with oral paracetamol and ibuprofen, unless contraindicated, and explicitly advised to take regular analgesia for a minimum of three days post-operatively.

### Surgical technique

On the day of surgery, prior to the start of the operative list, patients on the list underwent a world health organisation (WHO) style checklist with the rest of theatre and recovery team, and all high-risk patients were identified with a clear post-operative plan to manage them. Identification of pre-operative antibiotics, venous thromboembolism (VTE) prophylaxis, approximate length of the proposed procedure with any anticipated difficulties were also undertaken. Any anaesthetic or surgical concerns were highlighted to the rest of the team ensuring that all necessary theatre kits and accessories for a given case were available at hand.

A standard protocol-led procedure was carried out for every patient. Under general anaesthetic, patients had an initial cystoscopy and placement of a safety guidewire. A rigid ureteroscopy (4.5F or 6F Wolf or Storz rigid ureteroscope) was then carried out up to the renal pelvis or as far proximally as was safely achievable (or to the ureteric stone), and used to advance the second guidewire into the kidney. For renal stones, if appropriate, a ureteral access sheath (9.5F/11.5F or 12F/14F Cook Flexor sheath, size determined at the time) was introduced over a second guidewire to optimise visualisation, intrarenal pressure and to facilitate extraction of large or multiple renal stones. A flexible ureteroscopy (Storz FlexX2) and laser stone fragmentation was then carried out. All stones were managed by either laser fragmentation, basket extraction or a combination of the two, with all accessible stones fragmented to 1 or 2 mm or dust and larger fragments retrieved actively with a Cook Ngage stone extractor (Cook Medical, USA). In most cases, a 6F ureteral stent was placed post-URS and this was removed via a local anaesthetic flexible cystoscopy 1–3 weeks post-procedure. Unless clinically indicated, patients did not have a routine post-operative urethral catheter insertion and were discharged home the same day.

### Analysis of post-operative outcomes

Some cases of late completion of the URS procedure, defined as performed after 15:00 h, and/or those who underwent elective catheterisation post-procedure were admitted for overnight stay. This was to fit in with the closure time of our day-case ward at 20:00, and those patients anticipated to stay beyond this time were admitted electively for overnight stay and discharged the following morning. Patients were divided into four groups based on the LoS: day case (0 days), where patients were discharged the same day; <24 h, where patients were discharged within 24 h of having the procedure; 1–3 days, where patients were discharged between 1 and 3; and >3 days, where patients were discharged after 3 days (prolonged stay).

Reasons for failed DC-URS, complication rates, re-admission rates and date of discharge were determined from the electronic records. In patients where reason for failed DC-URS was multifactorial such as elective catheterisation followed by pain or sepsis, the predominant reason for prolonged stay was taken as the cause so that in the patient highlighted, this would be pain or sepsis.

Re-admission was defined as those patients presenting within 30 days of having had the procedure. Determination of total stone length has been previously described [11]—this was calculated by measuring the maximum stone diameter on a CT scan or, if multiple stones, the sum of the maximal dimensions of each stone. SFR was defined as complete disappearance or clinically insignificant fragments of stones 2 mm or less, as described elsewhere [12]. Post-operative stent removal under local anaesthetic was arranged in 1–3 weeks time and follow-up of patients was arranged at 2–4 months in a dedicated kidney stone clinic. All patients with radiopaque stone underwent plain radiograph and those with radiolucent stone an ultrasound scan of the kidneys, ureters and bladder to establish whether stone-free status was reached. Patients who were endoscopically and/or radiologically stone free at the end of the URS procedure (as recorded on electronic theatre notes) and were symptom free at stent removal with no re-admissions but did not attend follow-up were presumed be stone free.

## Results

### Day-case ureteroscopy

Between March 2012 and July 2016, 544 consecutive adult patients with stone disease underwent URS at our hospital, comprising 347 males and 197 females. Whilst the mean age was 56 ± 16.3 years (range 19–89 years), the mean stone size and the mean operating time were 14.2 mm and 46.6 min, respectively (Tables 1, 2). Stone characteristics, including location and size, are detailed in Table 2. The stone length was variable and tended to be larger for patients with longer hospital stay. Based on the LoS, the mean overall stone length varied between 14 and 18 mm. Approximately 26% (*n* = 141) of patients had stones in multiple locations and based on their LoS, it varied between 25 and 39% for different groups. Almost 58% (*n* = 315) of patients had renal stones, of which nearly half (*n* = 154) of them were in the lower pole. In a small number of patients (*n* = 19, 3%), no stone was seen during the operation (day case: *n* = 14; <24 h: *n* = 3; >3 days: *n* = 2) and the patients had either passed their stone or the stone was noted as a papillary calcification.

**Table 1:** Patient demographics and overall outcomes of the study

Patient demographics and clinical data (*N* = 544)	*n* (%) ± SD
Male:female (*n*)	347: 197
Mean age, years ± SD	56 ± 16.3
Mean length of stay (days) ± SD	0.49 ± 2.22
Day case	424 (77.9)
<24 h	72 (13.2)
1–3 days	23 (4.2)
>3 days	25 (4.6)
Positive pre-operative urine culture (treated appropriately), *n* (%)	56 (10.3)
Mean operative time (min) ± SD	46.6 ± 25.5
Access sheath, (%)	206 (38%)
Size: 9.5/11.5, 10/12	78 (14.4)
12/14	109 (20.2)
14/16	11 (2.0)
Not stated	8 (1.5)
Pre-operative stent, *n* (%)	153 (28.1)
Post-operative stent placement, *n* (%)	523 (96.1)
Stone-free rate, *n* (%)	472/501 (94.2)

**Table 2:** Stone parameters by length of stay (LoS)

LoS	Mean total stone length (mm) ± SD	No. of stones, *n* ± SD	Stone location, *n* (%)			
			Multiple locations	Renal	Ureteric	LP
Day case, *N* = 424	14.0 ± 15.3	1.86 ± 2.24	106/424 (25)	249/424 (58.7)	180/424 (42.5)	118/424 (27.8)
<24 h, *N* = 72	14.9 ± 10.9	1.88 ± 2.27	19/72 (26.4)	39/72 (54.2)	39/72 (54.2)	21/72 (29.2)
1–3 days, *N* = 23	13.8 ± 11.9	2.36 ± 2.08	9/23 (39.1)	17/23 (73.9)	9/23 (39.1)	10/23 (43.5)
>3 days, *N* = 25	18.1 ± 34.3	3.13 ± 7.5	7/25 (28)	10/25 (40)	9/25 (36)	5/25 (20)
		Total (%)	141/544 (25.9)	315/544 (57.9)	237/544 (43.6)	154/544 (28.3)

*LP* lower pole stone

423 of 544 (77.7%) patients were true day-case procedures (0 day) and a further 72 (13.2%) were discharged within 24 h. Only 25 (4.6%) patients in our cohort had a prolonged stay (defined as >3 days), as shown in Table 1. Fifty-six patients (10.3%) had a positive urine culture, which was treated appropriately prior to URS (Table 3). Mean operative time was 46.6 ± 25.5 min (range 8–152 min) and 96.1% of patients underwent post-operative stent placement. Mean operating time was significantly shorter for DCU compared to patients with longer stay (*p* < 0.02).

**Table 3:** Patient demographics and pre-operative characteristics by length of stay (LoS)

LoS	M:F	Mean age (years) ± SD	LoS (days), mean ± SD	Positive urine culture (treated appropriately), *n* (%)	Pre-operative creatinine (μmol/L), mean ± SD	Pre-operative stent, *n* (%)
Day case, *N* = 424	284:140	56.0 ± 16.0	N/A	32/424 (7.5)	87.5 ± 35.3	116/424 (27.4)
<24 h, *N* = 72	38:34	57 ± 18.4	N/A	12/72 (16.7)	101.3 ± 49.3	21/72 (29.2)
1–3 days, *N* = 23	10:13	57 ± 11.1	2.04 ± 0.71	6/23 (26.1)	88 ± 31.9	6/23 (26.1)
>3 days, *N* = 25	15:10	68 ± 15.1	8.76 ± 5.72	6/25 (24)	123.7 ± 85.8	10/25 (40)

### Failure of day-case ureteroscopy

Of the 121 patients that had in-hospital stay, the most common reasons for failure of day case were late completion of procedure (*n* = 24, 20%) and social factors such as living alone (*n* = 23, 19.2%) where they could not be safely discharged home the same day having had a general anaesthetic earlier (Table 4). In the 72 patients that were hospitalised overnight only, more than half (51.4%) were due to one of the above two reasons. Other commonly identified factors resulting in failure of day case were patients who were admitted as emergencies (*n* = 16, 13.3%) and elective post-operative catheterisation (*n* = 9, 7.5%). In the later cases, all patients were discharged following removal of catheter the next day. Aside from social factors and late completion of the procedure, we found seven cases of delayed discharge due to other non-urological factors, six of which resulted in prolonged hospitalisation of more than 3 days (Table 2). These factors included mental health issues necessitating in-patient psychiatry services and, in three cases, neuropathic leg pain, a cystic fibrosis patient with poor glycaemic control and problems with percutaneous endoscopic gastrostomy (PEG) feeding, respectively.

**Table 4:** Factors resulting in failed day-case URS by length of stay for all cases resulting in at least overnight admission (*n* = 121)

	<24 h, *n* (%)	1–3 days, *n* (%)	>3 days, *n* (%)
Factors resulting in failed day-case procedure (*n* = 99)			
Emergency admission with renal colic (*n* = 10)	8	2	
Emergency admission with urosepsis (*n* = 6)	2	3	1
Late completion of procedure (after 1500 h) (discharged the following morning) (*n* = 24)	24		
Social reasons (e.g. lives alone/frailty) (*n* = 23)	13	6	4
Anaesthetic decision for admission (e.g. epilepsy, CPAP dependent) (*n* = 3)	2	1	
Surgical decision for admission due to comorbidities (*n* = 5)	4		1
Surgical decision for admission due to previous history of urosepsis (*n* = 4)	1	2	1
Elective post-operative catheterisation (*n* = 9)	9		
Post-operative pain/stent-related discomfort (*n* = 8)	4	2	2
Other non-urological reasons (e.g. PEG feeding, cystic fibrosis, mental health issues, etc.) (*n* = 7)		1	6
Secondary to complications (22/544) (20—Clavien I–II, 1—Clavien III, 1—Clavien IV)			
a. Acute urinary retention	1	2	
b. Sepsis		1	8
c. Urinary tract infection		2	2
d. Ureteric perforation		1	
e. Haematuria (not requiring transfusion)	1		
f. Post-operative vomiting/dehydration	3	1	

*CPAP* continuous positive airway pressure

### Complications

Twenty-two (4%) patients in our study developed complications (Table 4). Of these, 20 cases of post-operative complications were all either Clavien grade 1 (*n* = 8, 36.3%) or grade 2 (*n* = 12, 54.5%). The most frequent post-operative complication was urosepsis (*n* = 9) and in patients with complications, this was the most common reason for prolonged stay. There was one Clavien grade 3 complication with small ureteric perforation recognised intraoperatively and treated with ureteric stenting. There was also one case of Clavien grade 4 complication where a patient with a background of multiple comorbidities including multiple sclerosis and recurrent urosepsis, developed candidaemia post-operatively, necessitating ITU admission, but made a full recovery. There was no mortality in our series. Although often presumed to be urosepsis, it should be noted that with the exception of the patient who developed candidaemia, discussed previously, no case of urine culture-proven source of infection was identified in the remaining eight patients.

### Re-admissions

There were 20 (4%) re-admissions from patients discharged within 24 h (Table 5). Seventeen of 423 (4%) patients who were day cases were re-admitted whilst the three re-admissions were those discharged within 24 h. The most common reason accounting for re-admission was post-operative pain or stent-related pain, none of who required more than overnight stay. In these cases, early stent removal was arranged. All cases of sepsis were managed with intravenous antibiotics and no case required ICU admission.

**Table 5:** Reasons for unplanned re-admission for all patients discharged within 24 h of ureteroscopy (*n*/*N* = 20/496)

Total number of patients re-admitted post-discharge <24 h (*N* = 496), *n* (%)	20 (4%)
Reason for re-admission	
Post-operative/stent-related pain	9
Urinary tract infection	4
Sepsis	5
Haematuria (not requiring transfusion)	2

Re-admission was defined as all unplanned admissions within 30 days of surgery

### Pre-operative, operative and stone characteristics

Despite appropriate prophylactic antibiotic treatment prior to their procedure, a significantly higher proportion of patients who were not able to go home on the day of their operation had a pre-operative positive urine culture (*p* < 0.001) (Tables 1, 3). Patients with prolonged hospital stay (>3 days) also had a significantly elevated pre-operative serum creatinine compared to the DC-URS (*p* < 0.001). Based on LoS, the mean age was also significantly higher for patients with prolonged hospital stay (*p* = 0.003).

Operative characteristics and SFR by LoS are shown in Table 6. Mean operative time, based on data available from 525 patients, was significantly shorter in the day-case group than all other groups (*p* < 0.001, compared to the prolonged stay group). Data on ureteral access sheath use were available for 540 patients, with an overall use in 38% of all patients. We did not have complete follow-up for 43 (7.9%) patients, either because follow-up occurred in a different geographical location or the follow-up is still pending having undergone URS relatively recently. Overall, 472 of 501 (94.2%) patients were found to be stone free at follow-up, with a SFR of over 95% for patients who underwent day-case ureteroscopy. Subsequently during further follow-up of these patients who were initially stone free, 23 (4%) of patients have had further treatment for recurrent stone disease.

**Table 6:** Operative characteristics and stone-free rate categorised by length of stay (LoS)

LoS	Mean operative time (min) ± SD	Access sheath, *n* (%)	Post-operative stent placement, *n* (%)	Stone free, *n* (%)
Day case	43.4 ± 24.1	162/421 (38.5)	409 (96.5)	372/390 (95.4)
<24 h	57.9 ± 26.7	25/72 (34.7)	68 (94.4)	59/67 (88.1)
1–3 days	60 ± 33.7	9/23 (39.1)	23 (100)	18/21 (85.7)
>3 days	54 ± 22.8	10/25 (40)	23 (92)	23/23 (100)
Overall		206/544 (38%)	523/544 (96%)	472/501 (94%)

## Discussion

### Meaning of study

With a rising incidence of stone disease, the role of URS in the day-case setting as a minimally invasive procedure is becoming more important [1]. However, despite a number of studies suggesting that it can be conducted in an outpatient setting, reports of its safety and efficacy as a true day-case procedure are limited [8–10, 13]. Studies with URS have predominantly been conducted in the setting of an in-patient hospitalisation or patients undergoing the procedure with overnight stay and therefore not true day cases [14, 15]. To our knowledge, no large series have investigated the outcomes and predictors for failure of day-case URS.

We report a high rate of successful day-case URS of 77.7% for consecutive adult patients, with a further 13.2% discharged within 24 h. Based on our study, patients with prolonged stay had significantly higher mean age, pre-operative positive urine culture, serum creatinine and operative duration compared to patients with DC-URS. Data from Hospital Episode Statistics (HES) show an overall increase in URS day-case rates over the last 8 years; however, they were still relatively low at 21 and 22% for renal and ureteric stones, respectively, for all procedures done in England (data from 2013 to 2014) [1].

### Unplanned re-admission and LoS <24 h

We found a low unplanned re-admission rate, with only 4% of day-case patients requiring unplanned re-admission. The most common reason for re-admission was secondary to post-operative pain or stent-related discomfort with all these patients requiring, at most, overnight admission for analgesia. In comparison, the CROES data on post-operative stent placement after ureteroscopy show a re-admission rate of 9 and 13% was seen for ureteric and renal stone treatment, respectively [16].

When excluding the delayed discharge for late completion of the procedure and social factors, the day-case rate for URS rose to 84.7%. Nevertheless, high day-case rate in our cohort of patients suggests that thorough pre-operative assessment, use of an anaesthetic protocol, procedure performed by an Endourologist and a dedicated day-case unit plays key roles in decreasing hospital stay. It also reiterates the importance of implementing anaesthetic and analgesia protocols, which have been conducted for other urological procedures such as retropubic prostatectomy to expedite discharge and reduce unplanned re-admission rates [17, 18].

### Emergency ureteric stones, stone parameters and day cases

Our study found that nearly a quarter of patients with ureteric stones (*n* = 57, 24%) were not done as day cases. These were predominantly cases where patients were admitted acutely and diagnosed with ureteric stone on CTKUB (non-contrast CT scan) and had an emergency ureteroscopy. To ensure their infection markers, renal function and/or symptoms had settled, they were observed and discharged having had their ureteroscopy and stone treatment. Of other parameters, we found that increasing age and multiplicity of stones appeared to be most consistently associated with greater LoS. Higher mean pre-operative serum creatinine and longer mean overall stone length were highest in the prolonged stay group (>3 days). With regards to stone parameters, previous studies have reported stone number and stone burden to be associated with higher complication rates post-procedure and this might partly explain our findings [19, 20]. In addition, we found that deployment of an access sheath was highest in the group with prolonged stay suggesting a more complex and larger nature of these stones. Taken together, these findings suggest that the more difficult ureteroscopic access was associated with increased LoS.

### Operating time, stone location and day-case rates

Unsurprisingly, mean operative time was shortest for the day-case group. However, the mean operative times of all other groups that were not done as day-case procedures were broadly similar between 54 and 60 min. Due to anatomical and technical factors, URS for lower pole stones compared to other locations tend to be more challenging [21, 22]. Of all the day-case procedures, 59% (*n* = 249) were renal stones consisting of 28% (*n* = 118) lower pole stones and 43% (*n* = 180) ureteric stones in multiple locations in 25% (*n* = 106) patients (Table 2).

In our results, we did not find a significant difference in the LoS for different stone locations (renal versus ureteric). Oitchayomi et al. recently reported that URS can be safely and effectively performed for upper urinary tract stones in the outpatient setting [15]. To date, studies have failed to show differences in complication rates for stone location post-URS [23–27]. With 58.7% of patients in our day-case group having renal stones and more than 25% of these located in lower pole, this study shows that patients with upper urinary tract stones can also undergo URS as true day-case procedures.

### Outcomes from this study compared to previous studies

We report an overall SFR of 94.2% after URS treatment at follow-up, a rate similar to previous studies [20] and a day-case SFR of 95.4%. We did not find a correlation between LoS and SFR, with both the day-case and prolonged admission groups producing excellent SFRs. Previous studies investigating URS in the outpatient setting have also reported an SFR exceeding 90% post-one session although studies have largely been retrospective with smaller sample sizes [8, 9]. It is also noteworthy that a standardised definition for stone-free status is currently lacking with SFR assessed at varying time points post-procedure [21].

### Post-operative stent insertion

Our study had overall post-operative stent insertion rates of 96%, which is higher than what has been previously reported [4, 28]. With our overall mean stone size of 14 mm, pre-operative stenting in 28% and access sheath use in 38%, renal stones were present in 58% and multiple stones in 26% of our patient cohort. When compared to CROES data for treatment of renal stones, 37% (*n* = 816) were pre-stented with overall post-operative stent insertion rates of 88% (*n* = 1963) and this was 91% for patients with the use of access sheath [28]. Although the overall stent insertion rates in CROES data (*n* = 11,885) were 81.5%, their data comprised 82% (*n* = 9681) ureteric stones of which 46% were in the distal ureter which had a substantially low post-operative stent insertion at 55% [4].

Stent insertion rates in our series reflect larger, perhaps more complex (renal, proximal ureteric or multiple) stones that were treated which is higher than reported in previously studies and raises a question of whether there is a price to pay for higher DC-URS rates. Clearly, a balance has to be struck between post-operative stent usage, unplanned re-admissions in patients without stents and quality of life in patients with stents. All efforts should be made to minimise post-operative stent usage whilst achieving high DC-URS rates.

### Strengths and limitations of the study

We advocate that a particular strength of this study is the availability of comprehensive prospective data for almost 550 consecutive patients spanning a period greater than 4 years at our institution. The unselected patient cohort includes regional referrals and complex stone patients, patients with a history of urosepsis and with a comparatively larger stone burden. Limitations of the current study include the absence of complete and long-term follow-up; thus we are unable to fully investigate the effect of LoS on recurrence rates requiring repeat sessions or other interventions. Future studies should evaluate data from URS procedures performed in a dedicated day-surgery setting, excluding diagnostic ureteroscopy, with time-independent discharge planning and a longer term follow-up. Perhaps, a comparison of reimbursement rates for ureteroscopy based on LoS in different healthcare systems and countries could explain variability in DC-URS rates and should be considered in the future.

## Conclusions

Ureteroscopy can be safely performed on a day-case basis for patients with renal and ureteric stones, provided there is no pre-operative positive urine culture or elevated serum creatinine, and the procedure has a relatively short operative time. The low rate of complications and unplanned re-admissions reiterates the success of ureteroscopy as a day-case procedure.
